# Heparin-based coacervate of bFGF facilitates peripheral nerve regeneration by inhibiting endoplasmic reticulum stress following sciatic nerve injury

**DOI:** 10.18632/oncotarget.18256

**Published:** 2017-05-29

**Authors:** Rui Li, Shuang Zou, Yanqing Wu, Yiyang Li, Sinan Khor, Yuqin Mao, Huacheng He, Ke Xu, Hongyu Zhang, Xiaokun Li, Jian Wang, Huai Jiang, Qike Jin, Qingsong Ye, Zhouguang Wang, Jian Xiao

**Affiliations:** ^1^ Molecular Pharmacology Research Center, School of Pharmaceutical Sciences, Wenzhou Medical University, Wenzhou, Zhejiang 325035, China; ^2^ The Institute of Life Sciences, Wenzhou University, Wenzhou, Zhejiang 325035, China; ^3^ Department of Peripheral Neurosurgery, The First Affiliated Hospital of Wenzhou Medical University, Wenzhou, Zhejiang 325035, China; ^4^ Department of Neonatology, The Second Affiliated Hospital of Wenzhou Medical University, Wenzhou, Zhejiang 325035, China; ^5^ Department of Molecular Pharmacology, Albert Einstein College of Medicine, Bronx, NY 10461, USA

**Keywords:** peripheral nerve injury, controlled release, basic fibroblast growth factor, endoplasmic reticulum stress, coacervate

## Abstract

Creating a microenvironment at the injury site that favors axonal regrowth and remyelinationis pivotal to the success of therapeutic reinnervation. The mature myelin sheath of the peripheral nervous system depends on active participation of Schwann cells to form new cytoskeletal components and tremendous amounts of relevant neurotrophic factors. In this study, we utilized a new biomaterial for growth factor delivery consisting of a biocompatible polycation, poly(ethylene argininylaspartatediglyceride) and heparin. It is capable of binding a variety of growth factors to deliver basic fibroblast growth factor (bFGF) through polyvalent ionic interactions for nerve repair. *In vitro* assays demonstrated that the bFGF loading efficiency reached 10 μg and this delivery vehicle could control the release of bFGF. *In vivo*, the coacervate enhanced bFGF bioavailability, which improved both motor and sensory function. It could also acceleratemyelinated fiber regeneration and remyelination and promote Schwann cells proliferation. Furthermore, the neuroprotective effect of bFGF-coacervate in sciatic nerve injury was associated with the alleviation of endoplasmic reticulum stress signal. This heparin-based delivery platform leads to increased bFGF loading efficiency and better controls its release, which will provide an effective strategy for peripheral nerve injury regeneration therapy.

## INTRODUCTION

Traffic accidents, trauma, and tumor resection can result in peripheral nerve injury (PNI). PNI is a common and serious health problem that can result in restricted activity and long-term disability. It was previously reported that about 3% of trauma patients are affected by PNI and require surgery [1]. At present, the gold standard treatment for peripheral nerve repair is autologous nerve grafting, but this technique is limited by tissue availability as well as immunological rejection [2]. Therefore, it is of great therapeutic interest to develop an alternative to the conventional grafting therapeutic method that provides a protected microenvironment that can spontaneously promote axonal sprouting and regeneration as well as supplements adequate growth factors (GFs) at the site of the lesion.

Neurotrophic factors (NTFs), including basic fibroblast growth factor (bFGF), have been widely used as a therapeutic strategy for preventing the PNI progression [3, 4]. bFGF is composed of 146 amino acids and belongs to a 22-member family of polypeptides. Recent advances inbiological materials have given way to a wealth of research about bFGF facilitating axon growth and nerve regeneration after PNI [5, 6]. However, bFGF has a very short half-life, and it is vulnerable to a variety of proteolytic cleavage events that would inactivate it in bodily fluids [7, 8]. Based on insufficient endogenous bFGF and its high affinity for heparin [9], we prepared a protein delivery coacervate to bind bFGF via charge interaction consisting of polycation-Poly(ethylene argininylaspartatediglyceride) (PEAD) and heparin [10]. This coacervate not only controls bFGF release but also maintains the bFGF's endogenous bioactivity [11]. Thus, this ternary complex could prove to be advantageous in treatments of complicated and refractory diseases including PNI.

Since the mechanisms of nerve regeneration are complex and not well understood, we wanted to explore how bFGF-Coacervate affects sciatic nerve injury. Endoplasmic reticulum stress (ERS) has been implicated in the pathogenesis of neurodegenerative disorders such as diabetic peripheral neuropathy (DPN), spinal cord injury (SCI), and cerebral ischemia-reperfusion (I/R) injury [12–14]. The endoplasmic reticulum (ER) is a membranous network in eukaryotic cells that functions in protein packaging and calcium storage [14]. Some of the events that lead to an imbalance between protein synthesis and protein folding can disrupt cellular homeostasis, leading to ERS [15]. To counteract ERS, cells activate the unfolded protein response (UPR) through a signal transduction cascade to restore ER homeostasis [16]. Because of the dearth of literature concerning the relationship between ERS and PNI, we sought to investigate this relationship and bFGF-Coacervate regulation.

**Figure 1:**
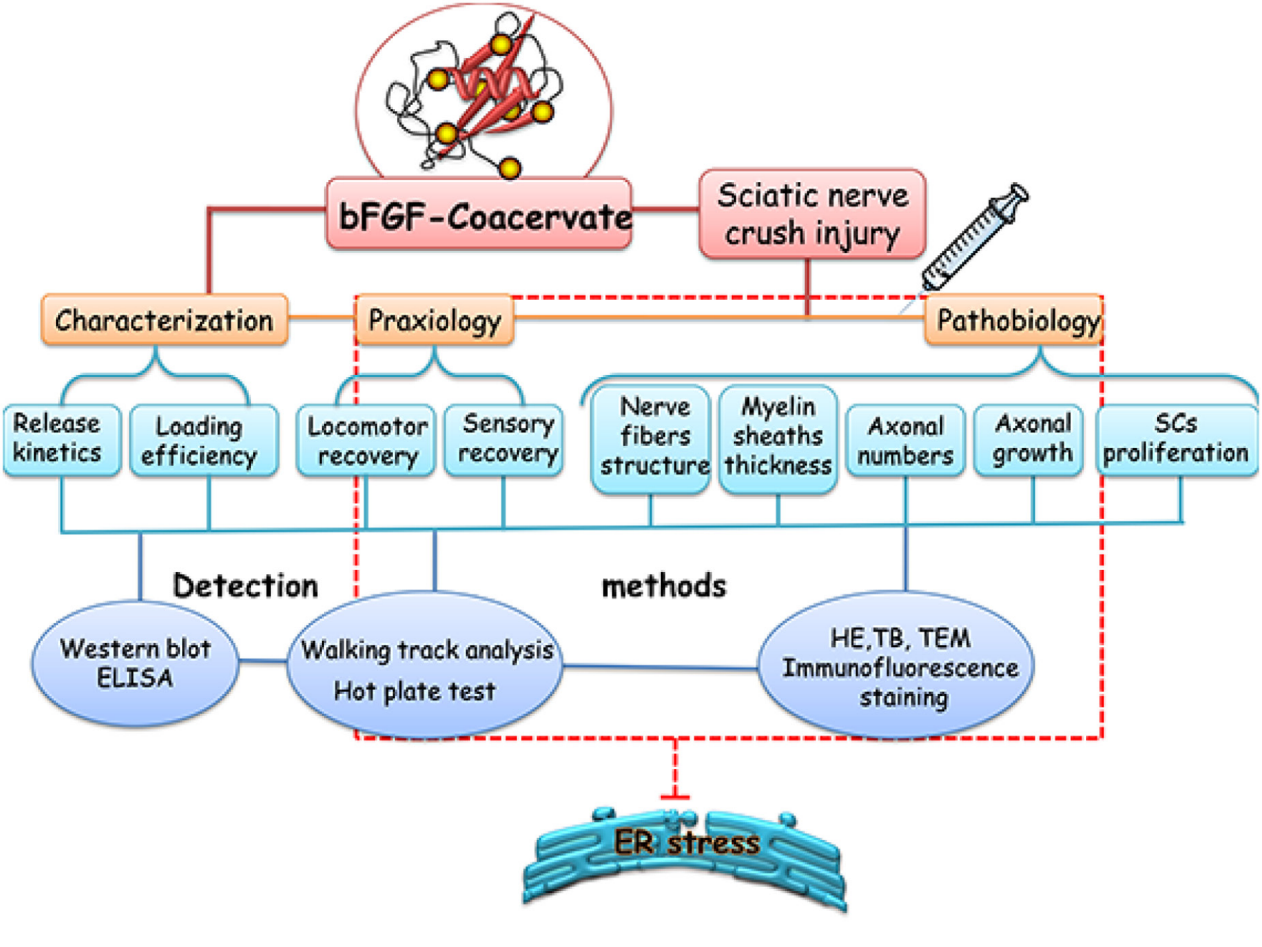
A schematic diagram of the experimental design We firstly prepared the [PEAD:heparin: bFGF] and detect its property through Western blot and ELISA methods. Next, this bFGF-coacervate was applied to repair the crush injury model of wistar rats via only one injection in the lesion region and analysis the functional improvement and nerve regeneration after 30 days recovery. Lastly, we demonstrated this excellent therapeutic effect was related to the inhibition of prolonged ERS in injuried tissue by Western blot.

Using the sciatic nerve crush injury model in rats, this work examined the beneficial effect of [PEAD:heparin] vehicle combined with bFGF on functional recovery and nerve regeneration. We also illustrated how the therapeutic effects of bFGF-Coacervate are mediated by the ERS-mediated axonal growth/atrophy signaling pathway. The detailed technology roadmap of this experimental design is shown in Figure 1. Overall, this study may have bearing on therapeutic strategies for promoting neural repair following PNI in clinical populations.

## RESULTS

### Characterization of bFGF-Coacervate

**Figure 2:**
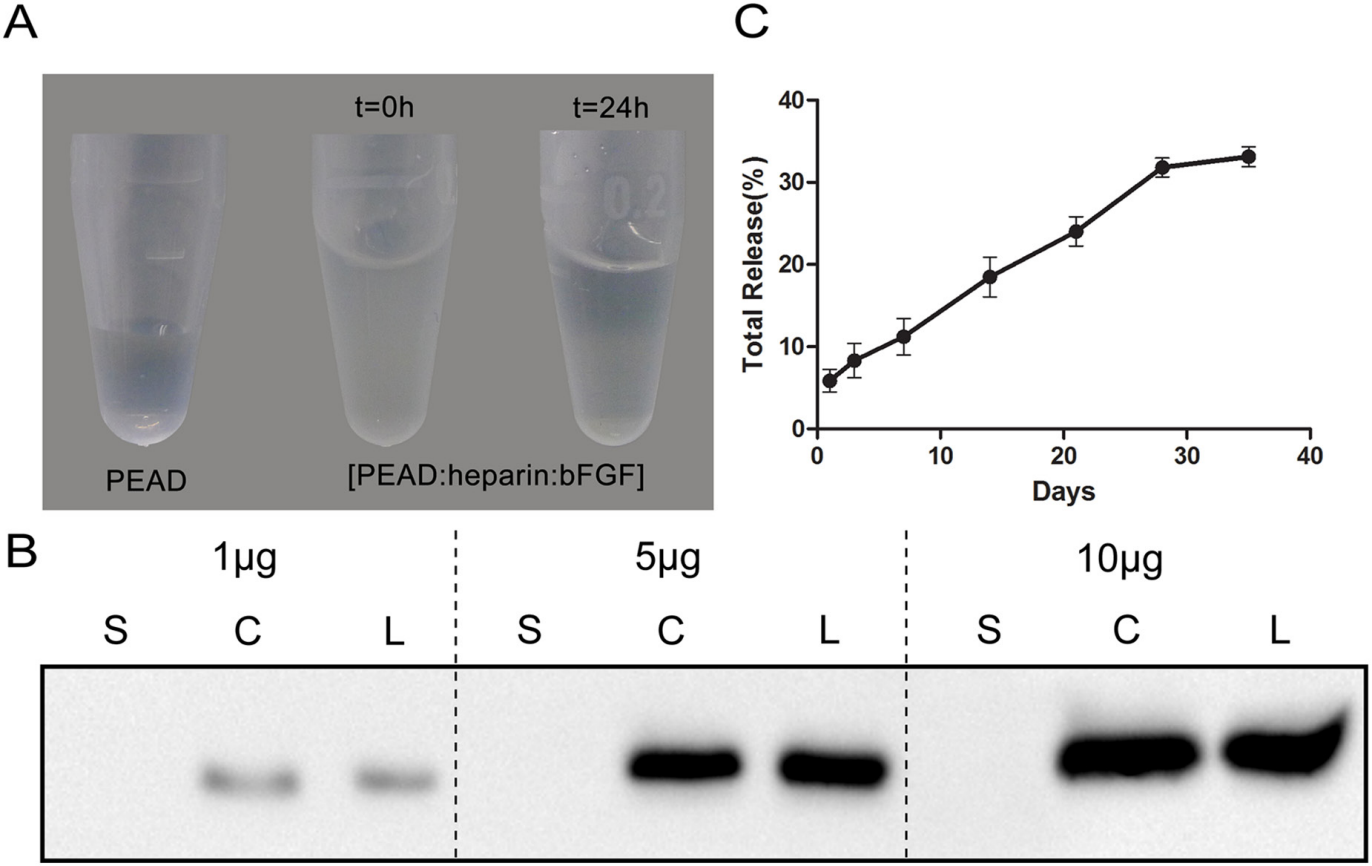
Properties of the [PEAD:heparin] vehicle binding to bFGF **(A)** In the preparation of bFGF-coacervate, the precipitation particles settle down to the bottom after 24 h standing. **(B)** Western blot analysis of the loading efficiency of bFGF into the coacervate (500 μg PEAD, 100 μg heparin, bFGF range tested: 1–10 μg). S: bFGF in the supernatant after centrifugation. C: bFGF in the settled coacervates. L: total amount of bFGF in the loading solution. **(C)** bFGF was released from the coacervate into saline at 25°C. The percentage of release over 35 days was quantified by ELISA. The data represent the means ± SD (n= 3 per group).

As a new delivery vehicle, [PEAD:heparin] interacts strongly with bFGF to form bFGF-Coacervate (or called [PEAD:heparin: bFGF] coacervate) through charge interactions [17]. This was visualized when the clarified heparin-bFGF solution became turbid upon addition of PEAD. However, after 24 h of standing, bFGF-Coacervateprecipitated down to the bottom (Figure 2A). It is known that the maximum binding mass ratio of PEAD/heparin is 5 by DMB assay [11], but the loading efficiency of [PEAD:heparin] binding to bFGF is unknown. To clarify this, 1 μg, 5 μg and 10 μg of bFGF were loaded into the [PEAD:heparin] matrix. Western blot results indicated that the amount of bFGF in the coacervate was almost the same as in the loading solution (Figure 2B) and thus, our choice of vehicle had a greater bFGF loading efficiency. We further collected the supernatantat indicated time points to determine bFGF concentration and draw the cumulative release profile of bFGF-Coacervate over 35 days (Figure 2C). There was a near linear, sustained release of bFGF for 28 days, after which it decreased and plateaued in the following days. At the end of the 35th day, approximately 60% of residual bFGF was unreleased. Overall, the release curve of bFGF–Coacervate indicated that this matrix can efficiently control the release of incorporated bFGF.

### bFGF-Coacervate promotes motor and sensory recovery after PNI

**Figure 3:**
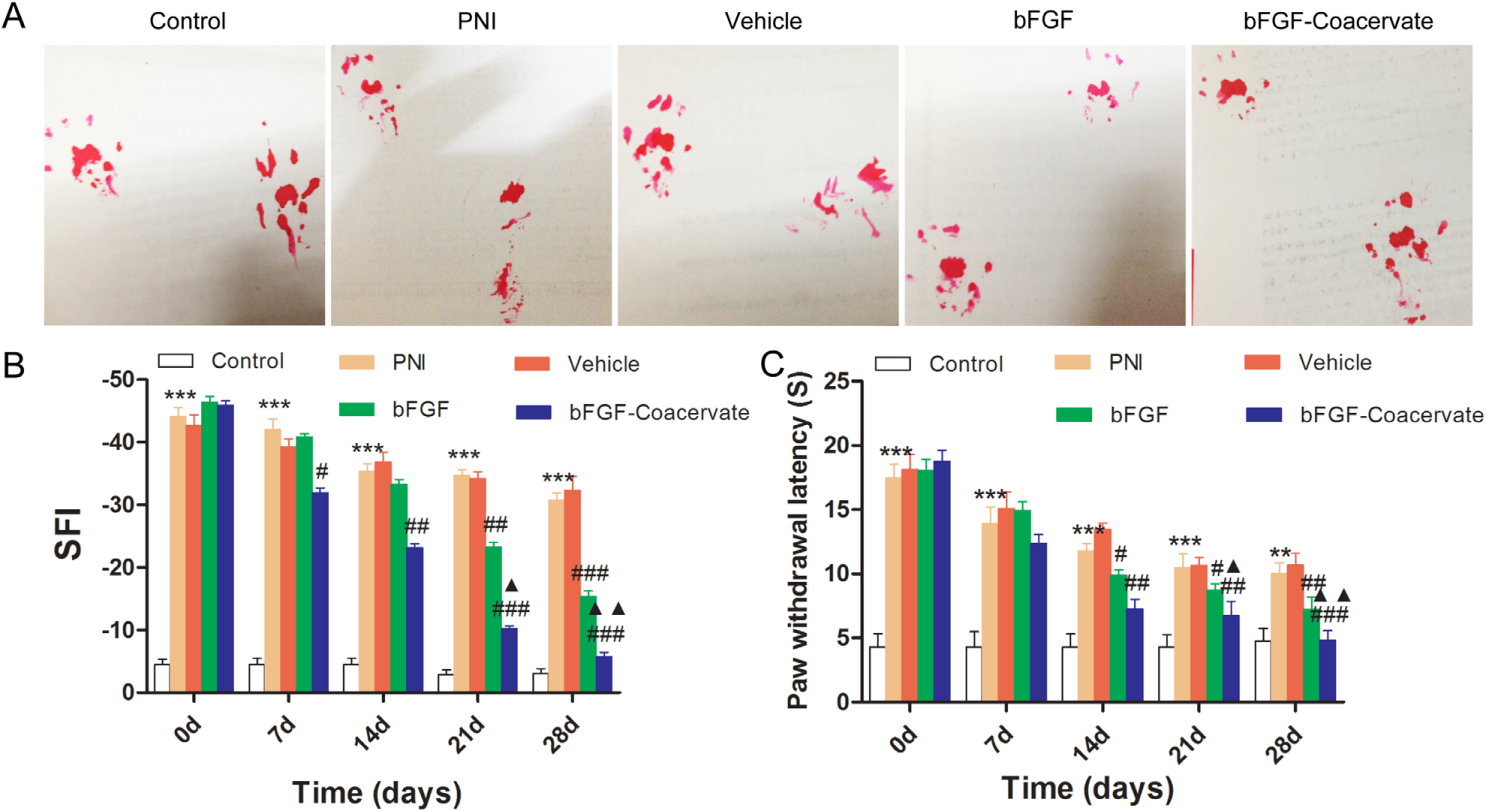
Locomotor and sensory recovery in each group were evaluated by walking track analysis and hot plate test **(A)** Representative pictures of rat footprint at 28 days of recovery after sciatic nerve crush. **(B)** Motor recovery was evaluated using the SFI analysis at 0, 7, 14, 21 and 28 days after surgery. **(C)** Statistical analysis of paw withdrawal latency before (0 day) and at indicated time points.^***^ P < 0.001 compared with control group, ^#^ P < 0.05, ^##^ P < 0.01,^###^ P < 0.001versus PNI group,^▲^P < 0.05, ^▲▲^P < 0.01 versus bFGF group.

We evaluated whether bFGF-Coacervate could promote motor and sensory recovery through out the 4-week period using the walking track analysis and hot plate test. The SFI value was not significantly different among the four crushed groups before week 2, although there was a trend toward a time-dependent increase. From week 3, the SFI value in each of the four crushed groups was significantly different. Locomotor performance in the bFGF animals was superior to the PNI and vehicle animals but inferior to the bFGF-Coacervate animals. Moreover, the superior recovery in the bFGF-Coacervate group continued through week 4 (Figure 3B, p < 0.01). Paw withdrawal latency as observed via the hot plate test showed nearly the same trend as the walking track analysis (Figure 3C). This demonstrates that bFGF-Coacervate can persistently improve the recovery of both motor and sensory function after crush injury, and the recovery effect is greater than routinely administrated bFGF.

### bFGF-Coacervate enhances myelinated fiber regeneration and remyelination at 30 days post-injury

**Figure 4:**
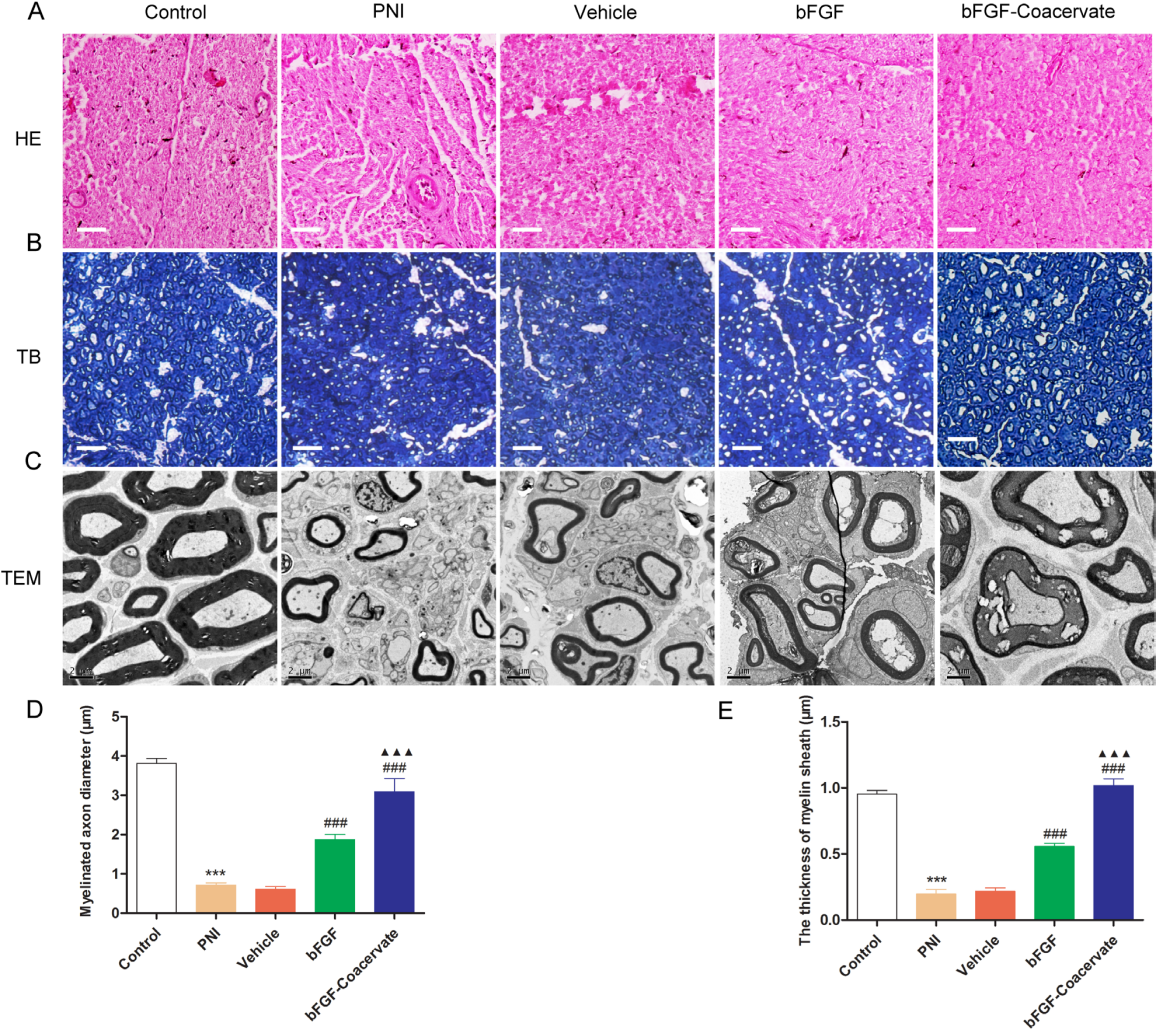
Histological investigation and morphometric analysis at 30 days after crush injury **(A)** H&E staining of sciatic nerve. Light micrographs of trans-sections in the five groups. Scale bar=50 μm. **(B)** Toluidine blue staining of semi-thin cross-sections from the distal end of crush nerve. **(C)** Electron microscopic images of the transverse sectioned injured nerves. **(D), (E)** Quantification of the myelinated axon diameter and the thickness of the myelin sheaths using the Image-Pro Plus software. ^***^ P < 0.001 compared with control group, ^###^ P < 0.001versus PNI group,^▲▲▲^P < 0.001 versus bFGF group.

To assess whether bFGF-Coacervate is capable of enhancing nerve fiber regeneration and remyelination, histomorphometrical analysis was performed at the distal portion of the injured site after 30 days restoration. H&E and TB sections showed that the regenerated nerve fibers in the bFGF-Coacervate and bFGF groups were denser, more compact, and uniform in comparison to the nerve fibers in the PNI and vehicle groups, which were small and irregular (Figure 4A and 4B). Importantly, we observed thin thickness and scattered density of regenerated myelin sheaths in the PNI and vehicle groups by TEM. Thicker, denser myelin sheaths were noted in the bFGF group, but myelin sheaths in the bFGF-Coacervate administration animals were the most thick and dense (Figure 4C). In addition, statistical analysis revealed that the mean axonal diameters and myelin thickness in with bFGF-Coacervate were the greatest (Figure 4D and 4E), although there was significantly statistical difference between bFGFgroup and vehicle groups as well. Taken together, these results show that bFGF-Coacervate had a beneicial effect on myelinated fiber regeneration and remyelination.

### bFGF-Coacervate accelerates axonal growth and promotes Schwann cell (SC) proliferation

**Figure 5:**
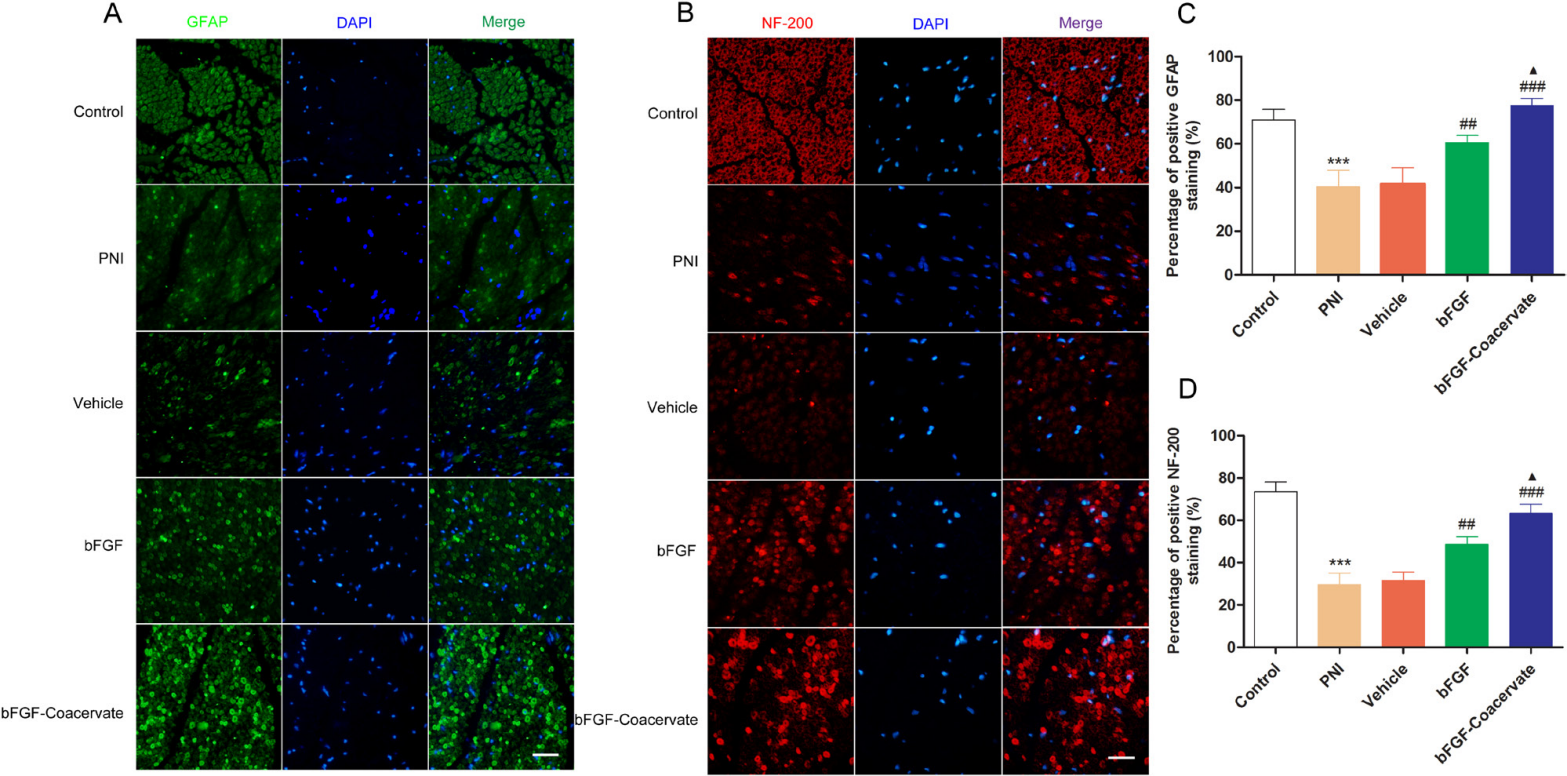
Evaluation of neurofilament-stained axonal growth and GFAP-positive SC proliferation in each group at 30 days post-injury **(A), (B)** Immunofluorescent staining of GFAP (green), NF-200 (red), DAPI (blue) and merging of the photographs (Scale bar=50 μm). **(C), (D)** Quantitative analysis of the GFAP and NF-200 positive staining areas in each group.^***^ P <0.001 compared with control group, ^##^ P < 0.01,^###^ P < 0.001versus PNI group,^▲^P < 0.05 versus bFGF group.

Following injury to a peripheral nerve, SCs undergo dedifferentiation and proliferation with the characteristic expression of glial fibrillaryacid protein (GFAP) [18]. NF-200 is the heavy subunit of neurofilaments for both larger and smaller axons. To further determine whether bFGF-Coacervate promotes SC proliferation and accelerates new axon growth, the expression of GFAP and NF-200 were examined by immunohistochemistry in injured sciatic nerve transections. As shown in Figure 4A and 4B, there was decreased GFAP and NF-200 signal in the PNI and vehicle groups. bFGF treatment could reverse these changes somewhat, but this increase was inferior to that with bFGF-Coacervate injection. Quantitative analysis also showed that tissue from the bFGF-Coacervate treated samples had significantly higher GFAP and NF-200 expression than the bFGF group (Figure 5C and 5D, p < 0.05). These results suggesting that bFGF-Coacervate increases the regenerative capacity of injured peripheral nerves.

### The regenerative effect of bFGF-Coacervate is mediated by inhibiting ERS after acute PNI

**Figure 6:**
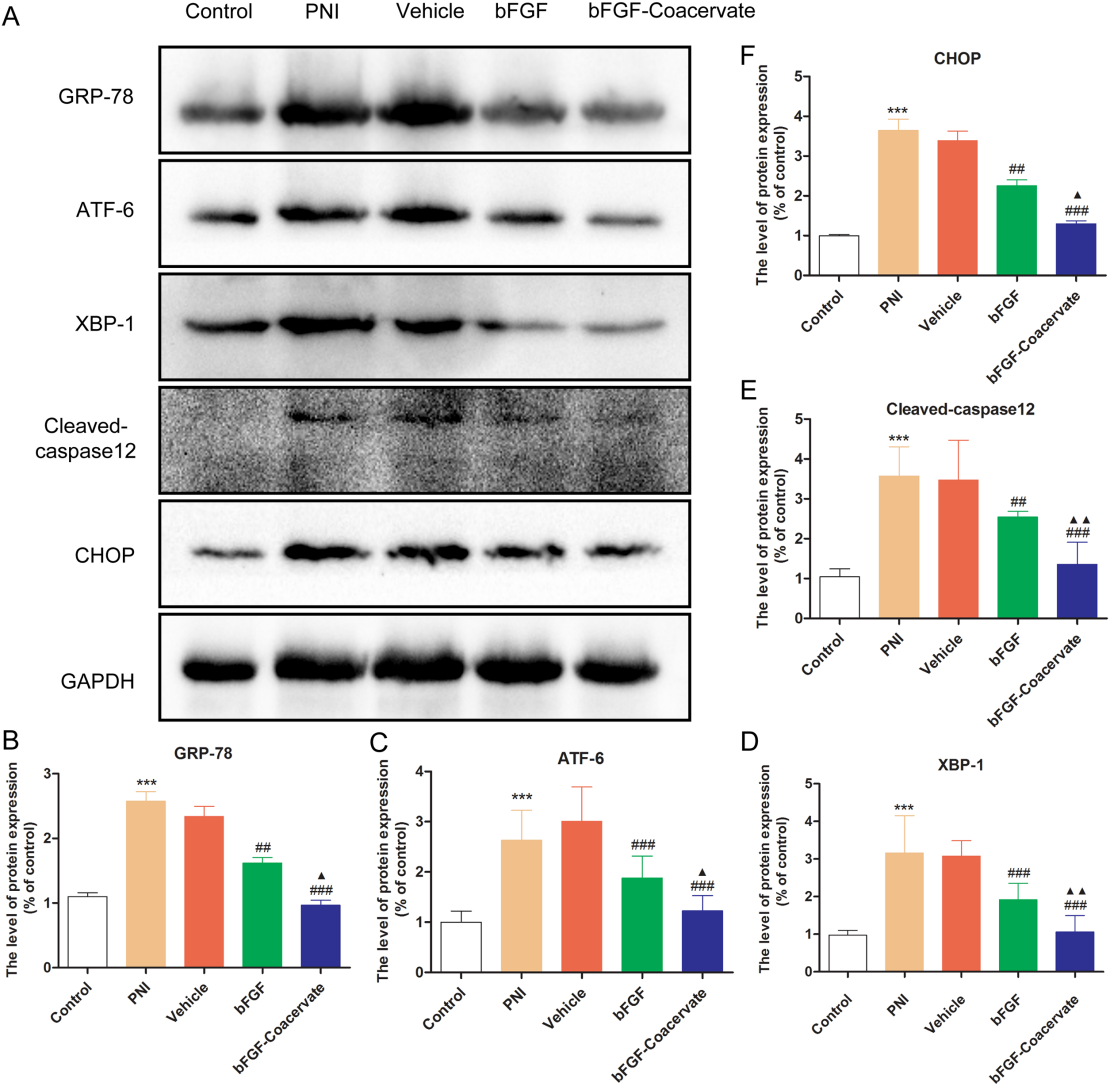
bFGF-coacervate administration reduced the contusion-induced ERS in injured sciatic nerve at day 30 following surgery **(A)** Immunoblot for GRP-78, ATF-6, XBP-1, Cleaved-caspase 12 and CHOP in each group after treatment with [PEAD:heparin], bFGF alone, or bFGF-coacervate. GAPDH served as a protein loading control. **(B-F)** The optical density analysis of GRP78, ATF-6, XBP-1, Cleaved-casepase-12 and CHOP proteins. ^***^ P < 0.001 compared with control group, ^##^ P < 0.01, ^###^ P < 0.001 versus PNI group, ^▲^P < 0.05, ^▲▲^P < 0.01 versus bFGF group.

It has been reported that excessive or prolonged activation of ERS can cause neuronal disorders, eventually leading to cellular apoptosis [16, 19]. However, it remains unclear whether ERS prevents the functional and structural recovery of an injured sciatic nerve and whether bFGF-Coacervate treatment can regulate ERS associated with PNI. These hypothesis in our study were detected by Western blot. As demonstrated in Figure 6, the levels of ERS-associated proteins GRP-78, ATF-6, XBP-1, Cleaved-caspase 12 and CHOP were increased significantly after 30d contusion. On the contrary, free bFGF combined with/without [PEAD:heparin] vehicle treatment could reduce the expression of these ERS markers. The bFGF-Coacervate samples displayed the greatest reduction in ERS markers. These changes were corroborated by statistical analysis of the band density (Figure 6B, 6C, 6D, 6E and 6F), which indicated that bFGF-Coacervate could suppress multiple components of the ERS pathway.

## DISCUSSION

Restoring the function of peripheral nerves following injury continues to be a challenging clinical problem. Currently, many protective materials and techniques are used to increase the success of nerve repair [20]. Artificial nerve conduits, typically consisting of biological materials filled with or without promoting elements, are comprehensively reported in rodent sciatic nerve injury model and human median nerve axotomy [21]. Silicone tubes, the first nerve conduits, are composed of nonresorbable polymers. They have been used to repair nerve injury for many years by increasing myelinated-axonal regeneration in an experimental 8 mm sciatic nerve gap model when filled with nerve growth factor (NGF) in the chamber [22, 23]. However, silicone tubes have been associated with some problems including nerve compression and requiring surgery to remove the resident conduits. Polyglycolic acid (PGA) is a highly biocompatible polymer and has been approved by the U.S. Food and Drug Administration (FDA) and Conformit Europe (CE) for use in tissue engineering. Wang et al. developed an artificial nerve graft which inserted longitudinal PGA filaments to bridge dog sciatic nerve transections with a 30-mm gap and achieved great therapeutic success [24]. However, PGA tubes are both expensive and difficult to handle during suturing, and they are only capable of bridging short nerve defects (nerve gap < 3 cm in length) [25, 26]. Current research has found that collagen conduits for nerve wrapping can sustain optimal axonal growth, nerve regeneration, and functional recovery due to their low antigenicity, excellent biocompatibility, and easy accessibility. It was shown that their scaffolds can be modified with bFGF and ciliaryneurotrophic factor (CNTF) to bridge a facial nerve injury creating a 35 mm gap in a minipig model. The technique enhanced facial nerve reconstruction and functional recovery 6 months post-surgery [27]. However, reports highlighting the lengthy degradation period of collagen conduits showed that they could lead to chronic nerve compression at later stages [28]. Thus, while current therapies exist to treat PNI, each has drawbacks or long-term consequences that warrant investigation for new therapies to treat PNI.

Therapeutic neurotization via exogenous NTFs including bFGF, nerve growth factor (NGF), and glial cell line-derived neurotrophic factor (GDNF) have been examined extensively to cure PNI [29–31]. In contrast to other NTFs that diffuse over relatively long distances [32], bFGF exhibits a variety of biological properties including neuroprotection, neurogenesis and angiogenesis in both the central nervous system (CNS) and peripheral nervous system (PNS). Previous research has demonstrated that endogenous bFGF increased the branching of transected axonsand promoted motor functionafter facial nerve cut [33, 34]. However, in this study, we focus on the effect of exogenous bFGF, and intrathecal administration of bFGF could markedly alleviate functional recovery after acute spinal cord injury (SCI) [35]. Fujimoto et al. applied exogenous bFGF for situ freeze-treatment in rat saphenous nerves and observed large numbers of regenerating axons by light and electron microscopy by day 5 [36]. Several other methods utilizing bFGF were developed to overcome its short half-life and ensure adequate bFGF delivery to injured areas. For instance, Ma et al. fused linear ordered collagen scaffolds filled with native bFGF to bridge a 5-mm rat sciatic nerve gap [6], Fujimaki et al. developed a new oriented collagen tubes combined with bFGF to repair 15-mm sciatic nerve transections in rats [37]. Both showed that the combination of bFGF with collagen enhanced axon regrowth and nerve regeneration over bFGF alone. However, whether the vehicles they used have high loading efficiency for NTFs, suitable biocompatibility, and enhanced bioactivity remains unknown.

To overcome the aforementioned challenges, we designed a new delivery vehicle-[PEAD:heparin]. In this solution system, PEAD is a polycation that can be easily synthesized, and its components including arginine, aspartic acid, glycerol, and ethylene glycol, are easily acquired. Heparin is well-studied glycosaminoglycan which can bind a variety of NTFs with high affinity. Thus, this delivery platform has the capability to conjugate bFGF by simple mixing them together. Furthermore, this ternary complex safe to use with no observable side effects, in part due to its low viscosity and good histocompatibility. In our previous study, we utilized this delivery platform to incorporate NGF, a neuroprotective factor, to repair the same crush injury model, and were able to achieve satisfactory repair as assessed by nerve regeneration and functional recovery [38]. In the present, such coacervate-bFGF binding helps to not only control the release of bFGF for 35 days but also load bFGF efficiently (Figure 2B and 2C). With moderate sciatic nerve contusion, only one injection of the bFGF-Coacervatewas required for sustained improvement of motor and sensory function (Figure 3). Treatment with bFGF-Coacervate exhibited the strongest myelinated fiber regeneration among all injury groups (Figure 4). bFGF-Coacervate also significantly increased axonal growth and SCs proliferation in comparison to free bFGF alone (Figure 5). These results suggested that bFGF-Coacervate could enhance the neuronal restoration seen with free bFGF administration. The difference between NGF-Coacervate and bFGF-Coacervate in promoting sciatic nerve recovery is due to the following two aspects. First, bFGF alone can promote nerve regeneration. However, its ability to enhance neovascularization has contributed to increased supply of nutrients and oxygen to the injury site. As a potent angiogenic factor, bFGF has a vital role in stimulating vessel sprouting and remodeling by recruiting vascular smooth muscle cells and pericytes [39, 40]. Furthermore, bFGF also triggers signaling of other angiogenic factors, which function to maintain the blood vessel networks [41], and we have demonstrated that this coacervate controlling bFGF release accelerates wound healing in full-thickness dermal wounds and maintains angiogenesis [42]. Thus, the protective effect of bFGF-Coacervate in nerve regeneration after injury is comprehensive including both neuroprotection and angiogenesis.

Accumulating evidence indicates that the functional recovery of bFGF after injury in both the PNS and CNS is related to altered levels of ERS. In our previous work, we demonstrated that the neuroprotective role of bFGF could modulated ERS in brain ischemia and reperfusion (I/R) injury and SCI [12, 43]. Here, we also found high levels of ERS marker proteins in the injured nerve at 30 days post-surgery, leading to poor remyelination and delayed locomotor recovery. This result is contrary to that found by Maritza et al [44]. They reported that activation of ERS promoted neural regeneration and functional recovery after PNI. This discrepancy may be explained in part by their focus mainly on the endoplasmic reticulum (ER) proteostasis network in the cellular changes at the early phase. However, apoptotic pathways can be activated with excessive or prolonged ERS. Thus, we observed scarce axon regeneration and little SC proliferation 30 days post-injury, but this could be reversed with administration of bFGF-Coacervate, which suppressed ERS protein expression (Figure 6).

In summary, we report that heparin based coacervate is a promising vehicle for enhancing bFGF bioactivity by promoting neuronal recovery and remodeling after sciatic nerve crush injury. Importantly, we have implicated the inhibition of prolonged ERS as a possible mechanism. Further study is warranted to translate this technology to the clinic and combine it with multiple proteins to enhance its therapeutic effect.

## MATERIALS AND METHODS

### Preparation of bFGF-Coacervate

The synthesis of Poly(ethylene argininylaspartatediglyceride) (PEAD) has been reported elsewhere [45]. First, ethylene glycol diglycidyl ether and t-BOC protected aspartic acid were polymerized through a polycondensation reaction in 1,4-dioxane to acquire the intermediate, poly(ethylene aspartate glyceride) (PED). Then, t-BOC and PED were coupled by a standard carbodiimide coupling reaction followed by the deprotection of t-BOC to obtain PEAD. To mark the controlled delivery matrix. PEAD, heparin, and bFGF were all 10 mg mL^-1^ after dissolved in saline. Next, heparin with or without bFGF was combined at 25°C for 15 min and then mixed with PEAD to obtain [PEAD:heparin:bFGF] (bFGF-Coacervate) or [PEAD:heparin]. The ratio of PEAD to heparin was 5:1 [11].

### bFGF-Coacervate loading efficiency

This method was conducted according to Chu et al [17]. First, heparin 100 μg and 1, 5 or 10 μg of bFGF were mixed followed by the addition of 500 μg of the PEAD solution (all solution concentrations are 10 mg mL^-1^). Upon gently mixing, bFGF was loaded into each coacervate. Then, the ternary complex was centrifuged at 12,000 rpm for 10 min. Both supernatant and precipitate as well as bFGF-Coacervate without centrifugation were mixed with the corresponding dose of 5×loading buffer and boiled at 100°C for 10 min. Finally, each solution was quantified by Western blotting and visualized by Quantity One® software.

### bFGF release kinetics

The release profile of bFGF from coacervate has been evaluated as described previously [42]. In brief, 0.5 μg of bFGF (10 ng μL^-1^), 10 μg of heparin (10 mg mL^- 1^), and 50 μg PEAD (10 mg mL^-1^) were combined. After centrifugation at 12,000 rpm for 10 min, the supernatant was collected and transferred to another Eppendorf tube. 500 μL saline (0.9%) was supplied at the same time on days 1, 4, 7,14, 21, 28, and 35. The amount of released bFGF in the collected supernatant was measured with the bFGF ELISA kit (Westang system, Shanghai, China) and absorbance was measured at 450 nm with a microplate reader.

### PNI model and drug administration

Male wistar rats (200~220 g) were obtain from the Laboratory Animals Center of Wenzhou Medical University. The living conditions and experimental procedures conformed to the National Institutes of Health (NIH) Guide Concerning the Care and Use of Laboratory Animals. All animal experiments described were approved by the Animal Experimentation Ethics Committee of Wenzhou Medical University, Wenzhou, China. All rats were kept in standardized laboratory conditions with regards to temperature (23±2°C), humidity (35−60%), and a 12:12 h light–dark cycle. Food and water were freely available to the animals for at least 7 days before the experiment.

Sciatic nerve injury was described previously [46]. Briefly, after anesthetizing with chloral hydrate (10%, 3.5 mL kg^-1^ body weight) through intraperitoneal injection, the right sciatic nerves in all experimental rats were exposed by blunt dissection from the biceps femoris space. At the site 7 mm proximal to the bifurcation, the sciatic nerve was crushed using two vascular clips (30 g force for 2 min, Oscar, China). The muscle layers and skin were sutured with a non-degradable suture. All rats were randomized into the following four crushed groups (PNI, vehicle, bFGF and bFGF-Coacervate) and a control group, n=8 for each group. The bFGF-Coacervate group was administered a single intramuscular orthotopic injection of [PEAD:heparin:bFGF] coacervate solution (300 μL) contained 15 μg bFGF, 750 μg PEAD and 150 μg heparin. Similarly, the vehicle group injected the same volume of bFGF-free coacervate solution. As for the bFGF group, these rats received 500 ng bFGF(dissolved in 100 μL saline) for 30 consecutive days. Given saline as control, other groups were given the same volume of saline. After 30 days, animals were sacrificed to collect the crushed nerve and assess the pathology index.

### Walking track analysis

To evaluate motor recovery in each experimental rat at 1, 7, 14, 21, and 28 days post-operation, walking track analysis was conducted by two independent examiners who were blinded to experiments. Individual footprints were recorded in the white paper after the rat walked through a 50cm×15cm×20cm glass box [47]. The changing parameters of paw prints were measured to calculate sciatic functional index (SFI) based on the following formula:

SFI = −38.3 × (EPL- NPL)/NPL + 109.5× (ETS - NTS)/NTS + 13.3 × (EIT - NIT)/ NIT-8.8

PL represents the distance between the third toe and heel, TS represents the distance between the first and fifth toes, IT represents the distance between the second and fourth toes, E is the injured right hind limb and N is the uninjured left hind limb. Generally, an SFI value of 0 represents normal nerve function, and SFI value around -100 indicates the sciatic nerve was crushed completely. SFI was a negative value and a higher SFI meant the better locomotive recovery of the sciatic nerve.

### Hot plate test

This experiment was performed by a single investigator who was blinded to the experiment to evaluate the sensory functional recovery by measuring the rat's ability to lick and shake its hind paw on the hot plate every week following drug administration. Before testing, each group was housed in individual plastic cages to accommodate the environment for 24 h. Then, the rats were placed on a hot plate (temperature: 55 ± 1°C). Next, the response time whenever the animals shaked or licked their paws was recorded. The cut-off time was set at 20s to minimize skin injury. All tests were repeated 4 times with 5 min intervals. If no hind paw withdraw was observed after 20 s, the response time was recorded as 20 s.

### Histological investigation and morphometric analysis

30 days post-surgery, animals were perfused with 4% paraformaldehyde in 0.1M phosphate-buffered saline (PBS), the sciatic nerve was isolated and rapidly excised at the injury sites and cut into two sections; one section (1-cm) was fixed in cold 4% (vol/vol) paraformaldehyde overnight and then dehydrated with gradient grade ethanol, embedded in paraffin, and cut into transverse paraffin sections (5-μm thickness) for hematoxylin and eosin (H&E) staining. The other segment (2-mm) was fixed in cold 2.5% (vol/vol) glutaraldehyde for 48 h and washed with PBS three times. The injured samples were immersed in 1% osmiumtetroxide solution and 1% uranyl acetate for 1 h, respectively, dehydrated in graded acetone series, and embedded in epoxy resin. Semi-thin sections (1-μm) from each segment were stained with toluidine blue (TB) and ultra-thin sections (50-nm) of three random areas were observed under a transmission electron microscope (TEM) (H-600, HITACHI, Japan). To analyze themorphometric data of the myelin sheath thickness, the diameter was measured with Image-Pro Plus software.

### Immunofluorescence staining

5-μm tissue sections in each group were dewaxed in xylene for 40 min and subsequently incubated in 3% H_2_O_2_ and 5% bovine serum albumin (BSA) for 30 min separately at room temperature. Sections were processed for labeling with antibodies against GFAP (1:100; Santa Cruz) and NF-200 (1:100,000; Abcam) overnight, followed by incubation with Alexa Fluor 488 or Texas Red conjugated secondary antibodies (1:1000; Abcam)) for 1 h at room temperature. Nuclei were labeled with 4′6-Diamidino-2-phenylindole-dihydrochloride (DAPI, Beyotime Institute of Biotechnology, Shanghai, China) [48, 49]. All photographs were taken at 400× magnificationusing a Nikon ECLPSE 80i microscope (Nikon, Tokyo, Japan).

### Western blot analysis

The contusion epicenter of sciatic nerve tissue (2 cm) was lysed in RIPA buffer containing phenylmethanesulfonyl fluoride (the volume ratio is 100:1) for 30 min and clarified by centrifugation at 12,000 rpm for 15 min. Then, the extracts were quantified with Carmassi Bradford reagents (Thermo, Rockford, IL, USA). 80 μg protein was separated using 12% sodium dodecyl sulfate polyacrylamide gel electrophoresis (SDS-PAGE) and electroblotted onto PVDF membranes (Millipore, USA). After blocking with 5% skim milk for 1.5 h, membranes were probed with primary antibody (GRP-78, ATF-6 and Cleaved-caspase 12, 1:1000, Abcam; XBP-1 and CHOP 1:300, Santa Cruz; GAPDH, 1:10000, Bioworld). Subsequently, blots were incubated for 1 h at room temperature with horseradish peroxidase-conjugated secondary antibodies (1:10000) [50, 51]. Finally, the immunoreactive bands were scanned via optical density measurements using the ChemiDoc XRS+ Imaging System (Bio-Rad). Each sample was blotted 3 independent times, and the densitometric values of the bands were quantified with the Image Lab software (Bio-Rad).

### Statistical analysis

All data are shown as the mean ± standard (SD). Statistical significance (defined as P < 0.05) was analyzed with GraphPad Prism 5 software (GraphPad Software Inc., La Jolla, CA, USA). Student's t-test was performed to compare the two experimental groups and one-way analysis of variance (ANOVA) with post hoc Dunnett's test was used to compare multi groups. For the walking track analysis and hot plate test, overall differences between groups were calculated using two-way ANOVA test.
